# miR-133a-3p/TRPM4 axis improves palmitic acid induced vascular endothelial injury

**DOI:** 10.3389/fphar.2023.1340247

**Published:** 2024-01-10

**Authors:** Yadong Xue, Tingting Tong, Yuyao Zhang, Haijun Huang, Ling Zhao, Hongzhao Lv, Lingzhao Xiong, Kai Zhang, Yuxuan Han, Yuyang Fu, Yongzhen Wang, Rong Huo, Ning Wang, Tao Ban

**Affiliations:** ^1^ Department of Pharmacology (The Key Laboratory of Cardiovascular Research, Ministry of Education, State Key Laboratory of Frigid Zone Cardiovascular Diseases, Ministry of Science and Technology) at College of Pharmacy, Harbin Medical University, Harbin, China; ^2^ Department of Anatomy, School of Basic Medical Sciences, Heilongjiang University of Chinese Medicine, Harbin, China; ^3^ Department of General Surgery, The Fourth Affiliated Hospital of Harbin Medical University, Harbin, China; ^4^ Heilongjiang Academy of Medical Sciences, Harbin, China; ^5^ National-Local Joint Engineering Laboratory of Drug Research and Development of Cardio-Cerebrovascular Diseases in Frigid Zone, The National Development and Reform Commission, Harbin, China

**Keywords:** vascular endothelial injury, hyperlipidemia, palmitic acid, TRPM4, MiR-133a-3p

## Abstract

**Background:** Vascular endothelial injury is a contributing factor to the development of atherosclerosis and the resulting cardiovascular diseases. One particular factor involved in endothelial cell apoptosis and atherosclerosis is palmitic acid (PA), which is a long-chain saturated fatty acid. In addition, transient receptor potential melastatin 4 (TRPM4), a non-selective cation channel, plays a significant role in endothelial dysfunction caused by various factors related to cardiovascular diseases. Despite this, the specific role and mechanisms of TRPM4 in atherosclerosis have not been fully understood.

**Methods:** The protein and mRNA expressions of TRPM4, apoptosis - and inflammation-related factors were measured after PA treatment. The effect of TRPM4 knockout on the protein and mRNA expression of apoptosis and inflammation-related factors was detected. The changes of intracellular Ca^2+^, mitochondrial membrane potential, and reactive oxygen species were detected by Fluo-4 AM, JC-1, and DCFH-DA probes, respectively. To confirm the binding of miR-133a-3p to TRPM4, a dual luciferase reporter gene assay was conducted. Finally, the effects of miR-133a-3p and TRPM4 on intracellular Ca^2+^, mitochondrial membrane potential, and reactive oxygen species were examined.

**Results:** Following PA treatment, the expression of TRPM4 increases, leading to calcium overload in endothelial cells. This calcium influx causes the assemblage of Bcl-2, resulting in the opening of mitochondrial calcium channels and mitochondrial damage, ultimately triggering apoptosis. Throughout this process, the mRNA and protein levels of IL-1β, ICAM-1, and VCAM1 significantly increase. Database screenings and luciferase assays have shown that miR-133a-3p preferentially binds to the 3′UTR region of TRPM4 mRNA, suppressing TRPM4 expression. During PA-induced endothelial injury, miR-133a-3p is significantly decreased, but overexpression of miR-133a-3p can attenuate the progression of endothelial injury. On the other hand, overexpression of TRPM4 counteracts the aforementioned changes.

**Conclusion:** TRPM4 participates in vascular endothelial injury caused by PA. Therefore, targeting TRPM4 or miR-133a-3p may offer a novel pharmacological approach to preventing endothelial injury.

## 1 Introduction

As the proportion of people on a high-fat diet increases, atherosclerosis has become a worldwide epidemic disease, accompanied by a high mortality rate (Herrington et al., 2016). When the levels of free fatty acids and cholesterol in the blood increase, a series of pathological changes occur in the vascular endothelium. The expression of cell adhesion and apoptosis molecules represented by VCAM-1 and ICAM-1 raises, inducing the endothelium to promote the accumulation of a large number of monocytes from peripheral blood (Malekmohammad et al., 2019). Research has found that increased levels of free fatty acids, represented by Palmitic Acid (PA), can cause endothelial cell apoptosis (Su et al., 2015; Chen et al., 2019). At present, the existing clinical strategies for the treatment of atherosclerosis have delayed the progress of atherosclerosis, improved arterial function, and decreased mortality rates, to a certain extent (Daiber et al., 2017). However, the current limitations of atherosclerosis treatment methods, such as large-scale clinical trials proving that the influence of antioxidants on arteriosclerosis progression have yielded disappointing results, which stemmed from there having been few tests of their beneficial effects on cardiovascular outcomes (Cook et al., 2007; Sesso et al., 2008). So, the key events of protecting the endothelium injury by hyperlipidemia remain to be explored.

The TRPM4 channel is a non-selective cation channel which is permeable to monovalent cations such as Na^+^ and K^+^. Research has shown that TRPM4 is generally expressed in tissues and organs such as arteries, veins, heart, and nervous system cells in the human body. During the occurrence of cardiovascular diseases such as arrhythmia (Guinamard et al., 2015), and myocardial remodeling (Wang et al., 2018), abnormal expression or sustained activation of TRPM4 is involved in the development of cardiovascular disease. *In vitro* experiments have shown upregulation of TRPM4 protein and mRNA expression levels in HUVECs under hypoxic conditions (Yao and Garland, 2005). Previous studies have clarified that TRPM4 was involved in developing myocardial hypertrophy (Watanabe et al., 2009). Overexpression of cardiac TRPM4 channels has been shown in spontaneously hypertensive rats (Abriel et al., 2012). However, there is still insufficient understanding of the role of TRPM4 in the process of vascular endothelial injury induced by hyperlipidemia.

microRNAs (miRNAs) play an irreplaceable role in the development of cardiovascular diseases. miRNA is a non-coding RNA with a length of approximately 20–22 nucleotides, which general binds to the 3′untranslated region (UTR) of the target mRNA and exerts inhibitory effects. miRNAs interfere with the pathophysiology of atherosclerosis by regulating atherosclerotic susceptibility genes and post-transcriptional gene expression (Lu et al., 2018). miR-1, miR-133, etc., play a role in vascular protection by regulating endothelial function (Gao et al., 2014; Navickas et al., 2016). Regrettably, the existing research has not yet provided a comprehensive explanation for the correlation between miRNAs and vascular endothelial injury.

The purpose of this research aims to investigate the possible involvement and mechanism of TRPM4 in the progress of endothelial cell damage triggered by PA. The main goal of this study is to establish a basic comprehension of the role that TRPM4 plays in the development of atherosclerosis resulting from hyperlipidemia.

## 2 Materials and methods

### 2.1 Antibodies and drug

The primary antibody against TRPM4 was obtained from Alomone (ACC-044, Jerusalem, Israel). Primary antibodies against BAX (WL01637), Bcl-2 (WL01556), and IL-1β (WLH3903) were purchased from Wanlei Biology (Shenyang, China). Primary antibodies against ICAM-1 (A5597), VCAM-1 (A0279), GAPDH (AC033), and β-actin (AC026) were obtained from abclonal (Wuhan, China). The secondary antibody (Dylight 800, Goat Anti-Rabbit IgG) was purchased from Abbkine (A23920, California, United States). Palmitic acid (PA) was purchased from Kunchuang Biotechnology (SYSJ-KJ003, Xi’an, China).

### 2.2 Culture of HUVECs

Human umbilical vein endothelial cells (HUVEC) cell lines (ATCC PCS-100-010TM) were obtained from the American Type Culture Collection (ATCC, United States), and cultured with the RPMI-1640 Medium (C11875500BT, United States) supplemented with 10% fetal bovine serum (04-010-1A, BI, Israel) and 1% Antibiotic-Antimycotic solution (C0222, Beyotime, China) at 37°C in a 5% CO_2_ incubator.

### 2.3 CCK8 assay

A 200 μL cell suspension containing 2 × 10^5^ cells per well was added to 96-well plates. The culture plate was then placed in an incubator at 37°C and 5% CO2 for 24 h. HUVECs were treated with varying concentrations of PA or a solvent control for 24 h. Following this, a 10 µL solution of CCK-8 (CA1210, Solarbio, Beijing, China) was added to each well, and the culture plate was again placed in the incubator for 30 min. The microplate reader was used to measure the absorbance at 450 nm.

### 2.4 Caspase-3 activity assay

Used the Caspase3 Activity Assay Kit (C1116; Beyotime) to perform Caspase3 activity assay. To start, lysed the treated cells using lysis buffer. Then, collected the supernatants of the homogenate by centrifugation at 12,000 g for 15 min and determined the protein concentration using a BCA protein assay kit. Next, incubated the cell lysates with Ac-DEVD-pNA (2 mmol/L) at 37°C for 2 h. Following the incubation, measured the absorbance at 405 nm using a microplate reader (BioTek).

### 2.5 qRT-PCR analysis

The HUVECs were seeded in a 6-well plate (2 × 10^5^/well) and stimulated with 0.2 mM PA or solvent control for 24 h. The total RNA of the cells was collected using the TRIZOL reagent (15596026CN, Invitrogen, United States) in accordance with the standard steps in the kit instructions. The cDNA was synthesized using the ReverTra Ace qPCR RT Master Mix kit (FSQ-201, TOYOBO, Japan), and the FastStart universal SYBR Green Master kit (Rox) (Roche, Switzerland) was used to amplify the cDNA. The primers used in qRT-PCR analysis are listed in Table 1. Target gene mRNA or miRNA expression was normalized to the expression of GAPDH or U6 and quantified utilizing the comparative 2^−ΔΔCT^ method.

**TABLE 1:** qRT-PCR Primer Sequence.

Gene-primer	Sequence
**miR-133a-3p**	F: 5′-TTT​GGT​CCC​CTT​CAA​CCA​GCT-3′
	R: 5′-ATC​CAG​TGC​AGG​GTC​CGA​GG-3′
**TRPM4**	F: 5′-GCA​CGA​CGT​TCA​TAG​TTG​ACT-3′
	R: 5′-CTT​CTC​CGT​GGT​GTG​TGC​AT-3′
IL−1β	F: 5′-TGA​GCT​CGC​CAG​TGA​AAT​GA-3′
	R:5′-CATGGCCACAACAACTGACG-3′
**ICAM-1**	F: 5′-TTG​GGC​ATA​GAG​ACC​CCG​TT-3′
	R: 5′-GCA​CAT​TGC​TCA​GTT​CAT​ACA​CC-3′
**VCAM-1**	F: 5′-GGG​AAG​ATG​GTC​GTG​ATC​CTT-3′
	R: 5′-TCT​GGG​GTG​GTC​TCG​ATT​TA-3′
**GAPDH**	F: 5′-CAT​GAG​AAG​TAT​GAC​AAC​AGC​CT-3′
	R: 5′-AGT​CCT​TCC​ACG​ATA​CCA​AAG​T-3′
β **-actin**	F: 5′-GGCTGTATTCCCCTCCATCH-3′
	R: 5′-CCA​GTT​GGT​AAC​AAT​GCC​ATG​T-3′
**BAX**	F: 5′-CCC​GAG​AGG​TCT​TTT​TCC​GAG-3′
	R: 5′-CCA​GCC​CAT​GAT​GGT​TCT​GAT-3′
**Bcl-2**	F: 5′-GGT​GCC​ACC​TGT​GGT​CCA​CCT-3′
	R: 5′-CTT​CAC​TTG​TGG​CCC​AGA​TAG​G-3′
**TRPM4-siRNA1**	GGA​GAU​AUG​GCC​AGC​ACU​ATT
	UAG​UGC​UGG​CCA​UAU​CUC​CTT
**TRPM4-siRNA2**	GGG​AGG​AGC​UAG​AGU​UUG​ATT
	UCA​AAC​UCU​AGC​UCC​UCC​CTT
**TRPM4-siRNA3**	CGU​GGG​AAU​CGG​UGC​AUA​ATT
	UUA​UGC​ACC​GAU​UCC​CAC​GTT

### 2.6 Western blot

RIPA Lysis Buffer (P0013B, Beyotime, China) was serviced to extract total proteins. After centrifuging at 13,500 rpm for 15 min at a 4°C centrifuge, the supernatant was gathered and determined by the BCA Protein Assay Kit (P0012, Beyotime, China). Equal solubilized proteins were separated on 10% polyacrylamide SDS gels and transferred onto a nitrocellulose membrane (PALL Gelman, United States). After blocked with 5% BSA, primary antibodies against TRPM4, BAX, Bcl-2, ICAM-1, VCAM-1, IL-1β (1:500), GAPDH (1:1000) were incubated with sample. After incubation in the 4°C refrigerator overnight, the NC membrane was incubated with the second antibody (1:7500) at room temperature for 50 min and was scanned by the densitometer utilizing the Odyssey infrared imaging system, and the fluorescence information was recorded.

### 2.7 Membrane protein expression assay

HUVECs were seeded in a 6-well plate and exposed to different concentrations of PA for 24 h. The cells were assimilated with 0.25% trypsin and centrifuged at 1,000 rpm for 5 min. The cells were resuspended with 200 μL of TRPM4 primary antibody dilution (1:200) and incubated for 1 h at room temperature. The cells were rinsed with 15 mL PBS and centrifuged at 1,000 rpm for 5 min. After incubation with 200 μL of secondary antibody diluent (1:4000) for 1 h at room temperature, the samples were cleaned by 15 mL PBS and centrifuged. Finally, the samples were resuspended in 500 μL PBS and detected by flow cytometry (Beckman Coulter, United States).

### 2.8 Electroporation transfection assay

2 × 10^5^ HUVECs were diluted in 400 μL of Opti-MEM Medium (31985070, Gibco, United States). Then, the diluted HUVECs were transferred into an electroporation cuvette with a 0.2 cm gap between the electrodes (1652092, Bio-rad, United States). Subsequently, 2 μL of 20 mM plasmid such as TRPM4 siRNA, TRPM4 plasmid, miR-133a-3p mimics, or negative control plasmid were implanted. The samples were electroporated using the Gene Pulser Xcell (165-2660, Bio-rad, United States) at 800 μF and 200 V. After electroporation, the samples were left at room temperature for 15 min. The cells that went through the aforementioned steps were then transferred to a cell culture plate and incubated for 24 h for subsequent experiments.

### 2.9 Annexin V/PI staining assay

HUVECs were plated at 2 × 10^5^cells per well in 6 well plates and exposed to 0.2 mM PA for 24 h. The cells were digested with 0.25% trypsin without EDTA and then cleaned twice with cold PBS and centrifuged at 1,000 rpm for 5 min. The cells were resuspended in 500 μL of 1× binding buffer at a concentration of 2 × 10^5^ cells/mL, 5 μL Annexin V-FITC and 10 μL PI were added (AT101, Multi Sciences, Hangzhou, China). Finally, the stained cells were harvested and analyzed utilizing flow cytometry (Beckman Colter, United States).

### 2.10 ROS levels assay

Using a DCFH-DA probe (S0033S, Beyotime, China), the cell ROS level was measured by confocal microscopy. HUVECs were plated on glass dishes with a 35 mm diameter and cultured at 37°C for 24 h. After treatment with 0.2 mM PA for 24 h, DCFH-DA solution (10 μM) was applied to each well and incubated for 30 min. The confocal microscope was used to measure the fluorescence at 525 nm, and ImageJ software was utilized to determine the formation of ROS.

### 2.11 Mitochondrial membrane potential (∆Ψm) assay

The cellular ∆Ψm change can be seen using the fluorescent probe JC-1. JC-1 forms red fluorescent polymers with wavelengths under 590 nm in the mitochondrial matrix of healthy cells. The mitochondrial membrane potential decreases in necrotic or apoptotic cells, and JC-1 primarily exists in monomeric form with green fluorescence at 530 nm. After rinsed the cells with PBS, the 24 well plates were implanted with the proper amount of JC-1 probe and incubated in the dark for 20 min. Red and green fluorescence intensity at 530 and 590 nm were measured by a laser confocal microscope, and the ratio of green to red fluorescence intensity was recorded and calculated.

### 2.12 Cellular calcium measurement

To measure intracellular calcium in HUVECs, this research used Fluo-4 AM, a cell-permeable fluorescent calcium indicator. Briefly, the cells were seeded at a density of 2 × 10^5^/well in 6-well plates and treated with PA for 24 h. The cells were cleaned with PBS three times and then stained with 2 μM Fluo-4 AM for 1 h a 37°C incubator. Intracellular calcium levels were evaluated by flow cytometry (Beckman Coulter, United States).

### 2.13 Luciferase reporter assay

A psiCHECK™-2 vector was made with a partial TRPM4 mRNA 3′-UTR including the miR-133a-3p target region (Promega, United States). Lipofectamine 2000 (Invitrogen, United States) transfection reagent was used to transfect into HEK293T cells with miR-133a-3p, miR-133a-3p inhibitor, or NC siRNA and psi-CHECK™-2-target DNA (firefly luciferase vector) or blank plasmid. A Dual-Luciferase reporter kit (Beyotime, China) was used to measure the luminosity of firefly and renilla luciferase following the manufacturer’s instructions. To adjust for discrepancies in transfection efficiency, the firefly/renilla ratio was determined.

### 2.14 Data Statistics

The standard error of the mean (SEM) is used to present experimental data. *p* < 0.05 was used to determine the significance of both the Student’s t-test and the one-way ANOVA for comparing two or more groups. Both tests were done using the analytic application GraphPad Prism 9.

## 3 Results

### 3.1 PA induces apoptosis and inflammatory cytokines expression in HUVECs

**FIGURE 1:**
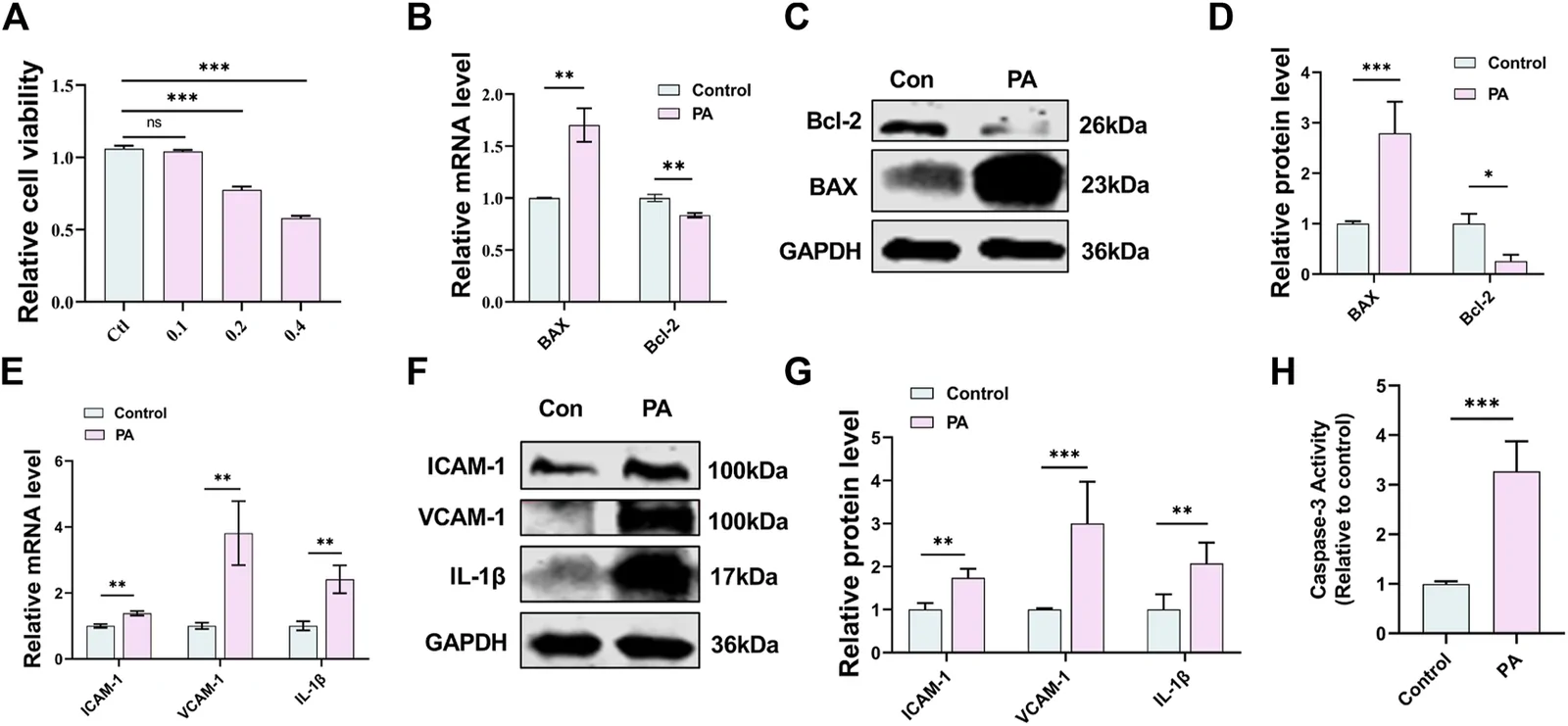
PA induces apoptosis and inflammatory cytokines expression in HUVECs. **(A)** After treatment with vary concentrations PA or solvent control for 24 h, the viability of HUVECs was assessed with CCK8 assay. **(B–D)** qRT-PCR and Western blot analysis of BAX and Bcl-2 expression in HUVECs after treatment with 0.2 mM PA or solvent control for 24 h **(E–G)** qRT-PCR and Western blotting analysis of ICAM-1, VCAM-1, and IL-1β mRNA and protein expression. **(H)** Caspase-3 activity assay kit measure of Caspase-3 activity after treatment with 0.2 mM PA. Data are expressed as the mean ± SEM, *n* = 3 in each group, *p < 0.05, **p < 0.01, ***p < 0.001 relative to respective control groups.

Previous studies have demonstrated that endothelial injury occurs in the initial stages of atherosclerosis, which is accompanied by an upregulation in the expression of inflammatory cytokines by the endothelium (Xu et al., 2021). High fat diet will exacerbate the level of free fatty acids in the blood, and then induce lipid metabolism syndrome, such as obesity, nonalcoholic fatty liver, atherosclerosis, etc. These chronic diseases can induce inflammatory reaction and detriment endothelial function (Mei et al., 2016). Palmitic Acid (PA), as one of the most common serums FFA, was wielded to treat cells in our research. HUVECs were handled with PA for 24 h *in vitro* to mimic the vascular endothelial injury by hyperlipidemia. Follow the concentration of PA increases, cell viability was gradually decreased. It was observed that the viability significantly decreased at concentrations of 0.2, and 0.4 mM PA compared to the solvent control group (Figure 1A). Considering the harmful effects of high PA concentrations, 0.2 mM was selected for subsequent testing. BAX mRNA level was found increased and Bcl-2 mRNA level was decreased in the qRT-PCR results after PA treatment (Figure 1B). At the protein level, Western blot results revealed that PA facilitated BAX expression while reducing Bcl-2 expression (Figures 1C, D), corroborated the accelerating effect of PA on HUVECs apoptosis. Moreover, ICAM-1, VCAM-1, IL-1β mRNA, and protein levels were visibly uplifted in HUVECs after treatment with PA (Figures 1E–G). Next, we measured the activity of Caspase-3 using a Caspase-3 activity assay kit. Treatment with PA significantly increased Caspase-3 activity (Figure 1H). The results indicated that PA induces apoptosis of HUVECs and promotes the release of inflammatory factors.

### 3.2 PA induces upregulation of TRPM4 in HUVECs

**FIGURE 2:**
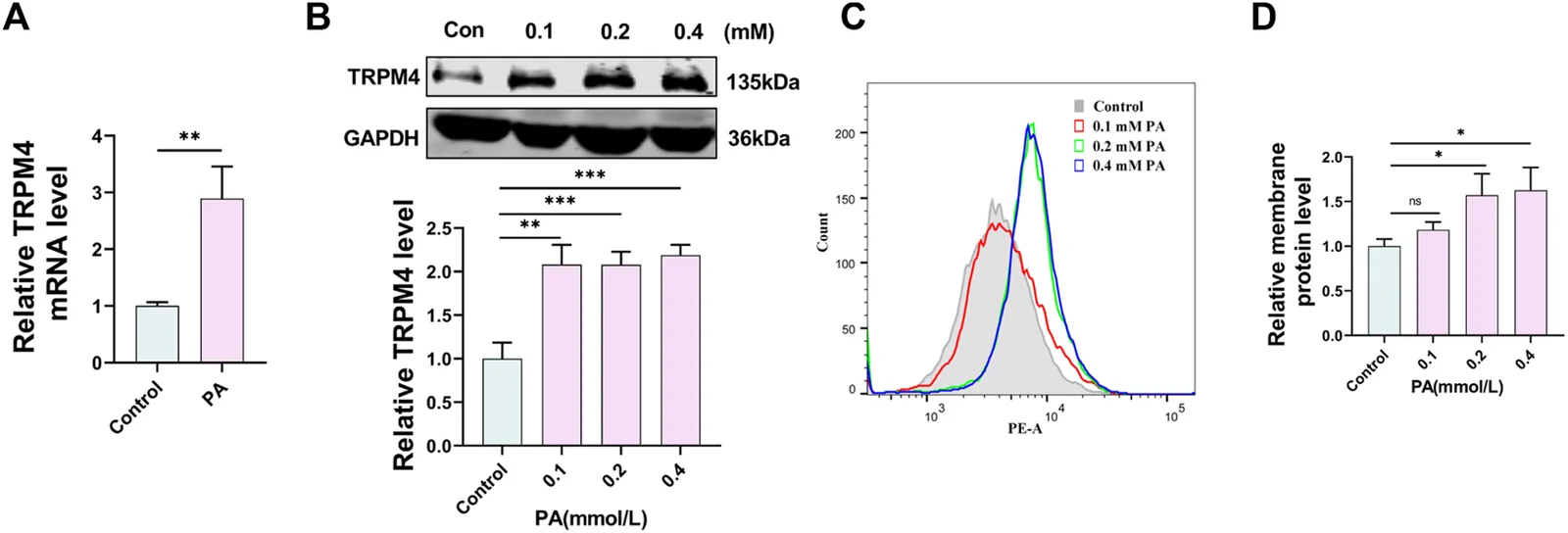
PA induces upregulation of TRPM4 in HUVECs. **(A)** qRT-PCR analysis of TRPM4 mRNA expression in HUVECs after treatment with 0.2 mM PA or solvent control for 24 h. **(B)** Western blot analysis of TRPM4 protein expression in HUVECs after deal with 0.1, 0.2, 0.4 mM PA or solvent control for 24 h. **(C,D)** Analysis of TRPM4 membrane protein by flow cytometry. Data are expressed as the mean ± SEM, *n* = 3 in each group, *p < 0.05, **p < 0.01, ***p < 0.001, relative to respective control groups.

It has been shown that TRPM4 was participate in the progression of endothelial injury (Dienes et al., 2021). However, its role in the treatment of endothelial injury in PA is unclear. Compared to the control group, the groups treated with PA showed increased levels of TRPM4 mRNA and protein levels as depicted on the Western blot and qRT-PCR (Figures 2A, B). Further study revealed that as the PA concentration increased, the level of TRPM4 protein on the cell membrane also significantly increased, in line with the change in total protein level (Figures 2C, D).

### 3.3 Knockdown of TRPM4 alleviates PA-induced apoptosis and inflammatory cytokines expression

**FIGURE 3:**
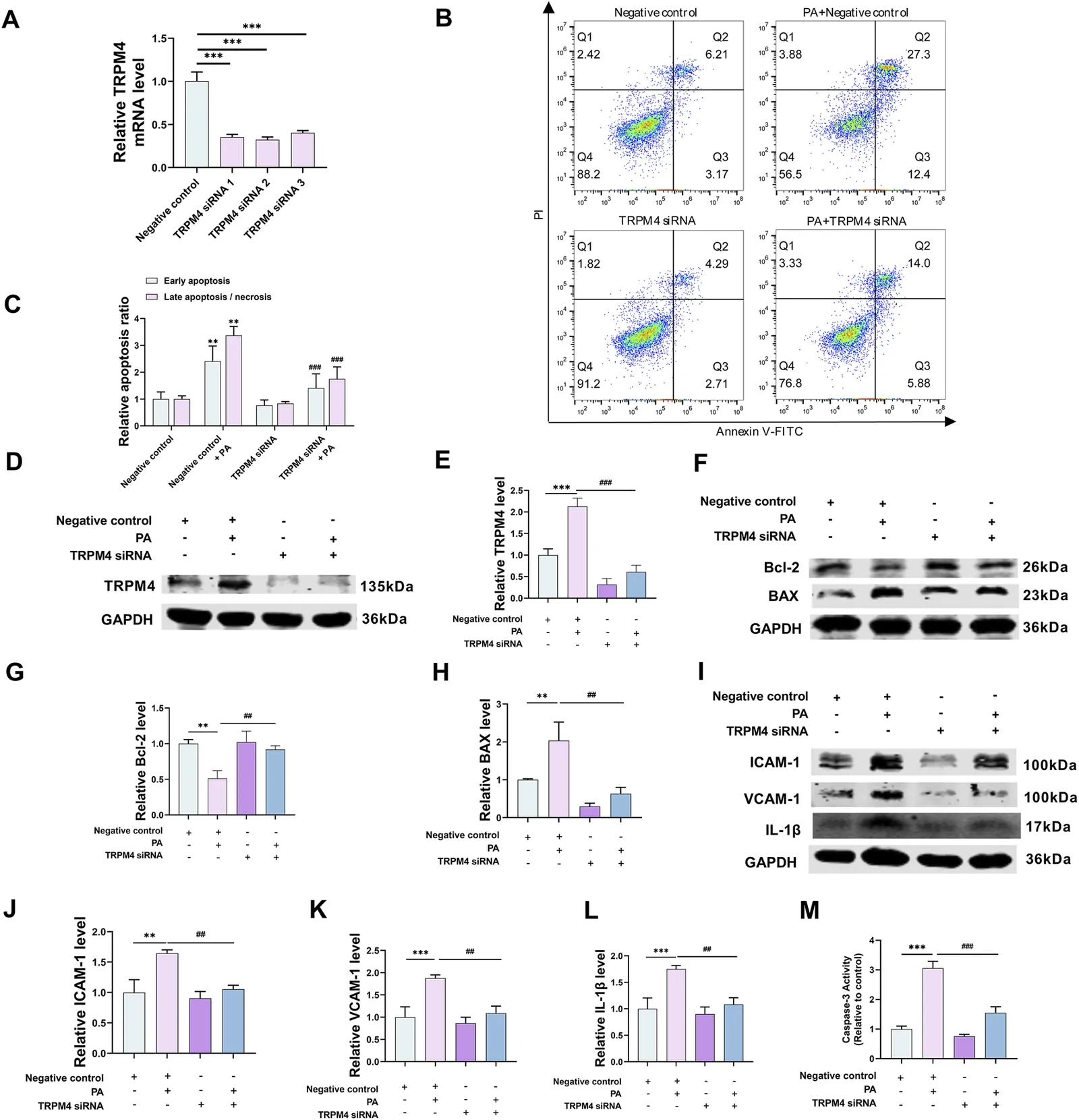
Knockdown of TRPM4 alleviates PA-induced apoptosis and inflammatory cytokines expression. **(A)** HUVECs knockdown TRPM4 efficiency evaluated by qRT-PCR. HUVECs were transfected with TRPM4 siRNA one or its negative control and exposed to PA for 24 h or solvent control for subsequent experiments. **(B, C)** Annexin V/PI staining assay was used to detect apoptosis. **(D–L)** Western blotting analysis of TRPM4, BAX, Bcl-2, ICAM-1, VCAM-1, and IL-1β protein expression and their quantitative statistics. **(M)** Caspase-3 activity was shown in the different groups. Data are expressed as the mean ± SEM, *n* = 3–4; in each group, **p < 0.01, ***p < 0.001 vs. negative control; ^##^ *p* < 0.01, ^###^ *p* < 0.001 vs. negative control + PA.

To study the biological role of TRPM4 in endothelial cells in a lab setting, HUVECs were altered with TRPM4 siRNA to decrease its expression. TRPM4 mRNA levels were noticeably reduced by TRPM4 siRNA. There was no notable variance among the three sequences of TRPM4 siRNA, hence, TRPM4 siRNA 1 was chosen for the following experiments (Figure 3A). Annexin V-FITC and PI assay were used to examine the cell apoptosis. The apoptosis ratio was significantly decreased in cells transfected with si-TRPM4 compared with the NC group with PA treatment (Figures 3B, C). Without PA treatment, TRPM4 knockdown did not affect the apoptosis of HUVECs. Interestingly, PA affected not only early apoptosis but also late apoptosis/necrosis, and TRPM4 knockdown after PA stimulation relieves both early apoptosis and late apoptosis/necrosis (Figures 3B, C). Meanwhile, the abundance of TRPM4 protein levels was significantly increased with PA treatment, which was restored by TRPM4 knockdown (Figures 3D, E). Moreover, Western blot indicated the promotion of Bcl-2 and reduced BAX protein level in the si-TRPM4 group compared with the NC group with PA treatment (Figures 3F–H), confirming the safeguard function of si-TRPM4. The Bcl-2 family influences apoptosis by managing the permeability of the mitochondrial outer membrane (Siemen and Ziemer, 2013). TRPM4 could also have an impact on apoptosis via the mitochondrial pathway, this part will be introduced in detail later. Meanwhile, the abundance of ICAM-1, VCAM-1, and IL-1β protein levels were significantly raised with PA treatment, which were restored by TRPM4 knockdown. Similar findings were noted when detecting the activity of Caspase-3. In addition, TRPM4 knockdown did not affect apoptosis and inflammatory cytokine expression without PA treatment (Figures 3I–M).

These findings collectively imply that the reduction of apoptosis and the suppression of inflammatory cytokines release in PA-induced endothelial injury is due to the silence of TRPM4.

### 3.4 Knockdown of TRPM4 protects HUVECs via mitochondrial pathway

**FIGURE 4:**
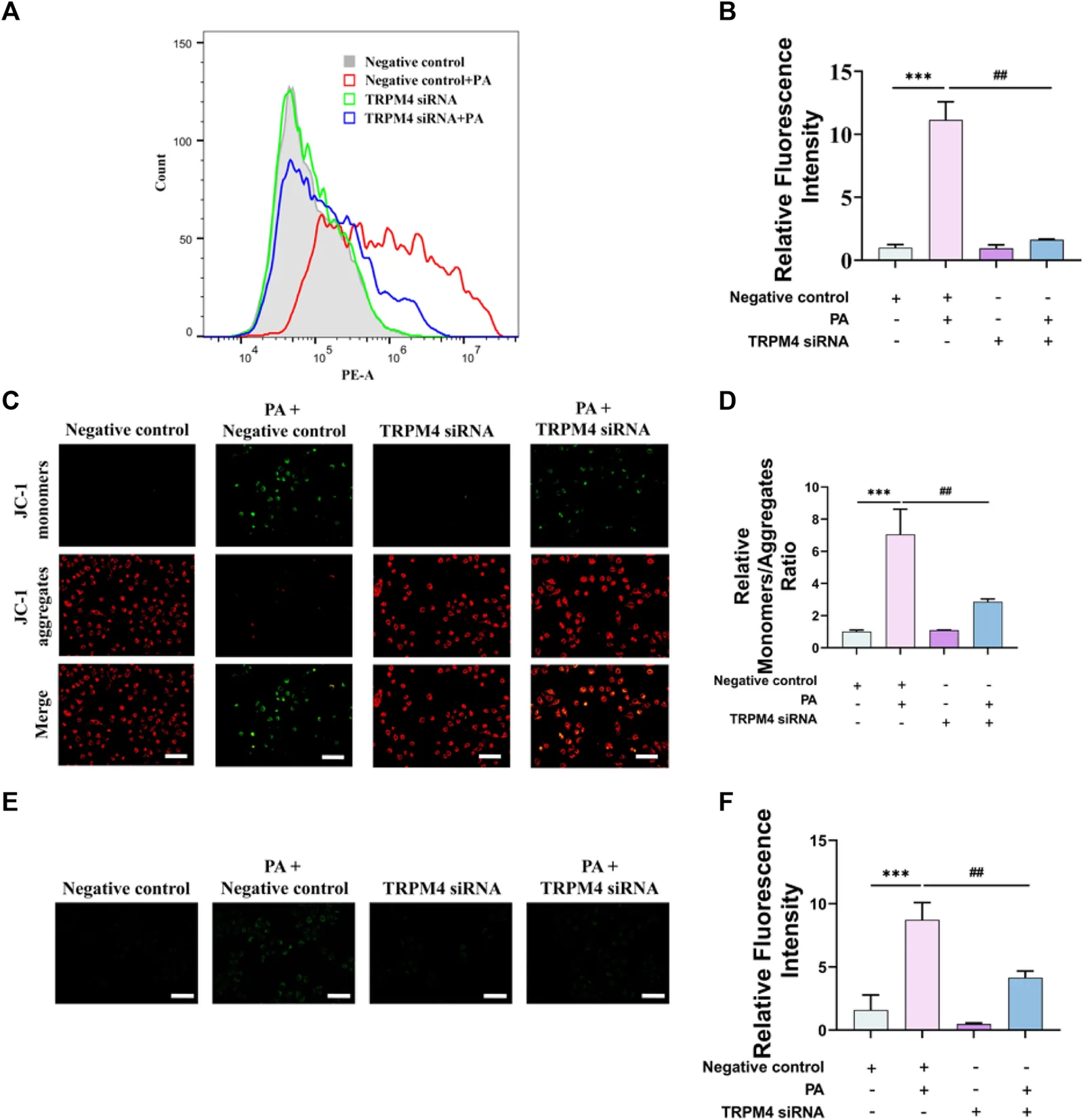
Knockdown of TRPM4 protects HUVECs via the mitochondrial pathway. HUVECs were transfected with TRPM4 siRNA or its negative control and then exposed to 0.2 mM PA or solvent control for 24 h **(A, B)** The relative fluorescence intensity of intracellular calcium was displayed by Fluo-4 AM through flow cytometry. **(C)** Fluorescence microscopy analysis of the level of JC-1 monomers (green fluorescence) and J-aggregate forms (red fluorescence). Scale bar, 50 μm. **(D)** The quantified relative green *versus* red fluorescence values. **(E)** Fluorescence microscopy analysis of the level of cellular ROS. Scale bar, 50 μm. **(F)** The quantified relative fluorescence intensity. Data are expressed as the mean ± SEM, *n* = 3–4; in each group, ***p < 0.001 vs. negative control; ##*p* < 0.01 vs. negative control + PA.

Our previous research has clarified that the upregulation of TRPM4 caused the accumulation of ROS and promoted cell apoptosis (Yu et al., 2019). To confirm if calcium and oxidative stress play a role in PA-induced endothelial injury, with TRPM4 serving as an immediate receptor potential channel, Fluo-4 AM was serviced to label intracellular calcium. It was found that si-TRPM4 with PA group showed a less average fluorescence intensity compared to the PA group (Figures 4A, B). The mitochondrial membrane potential (∆Ψm) was assessed using fluorometric analysis after staining with JC-1. Green *versus* red fluorescence values were wielded to reflect the degree of mitochondrial damage. The TRPM4 siRNA add PA group had a lower percentage value of fluorescence intensity of green *versus* red fluorescence compared with the negative control with PA group (Figures 4C, D). In the bargain, the production of ROS was examined using a DCFH-DA assay kit via a fluorescence microscope. With PA treated, the cellular DCF fluorescence intensity was lower in cells transfected TRPM4 siRNA than that of cells transfected negative control (Figures 4E, F).

The results indicated that mitochondria injury and ROS accumulation in HUVECs were protected by knockdown TRPM4. Consistent with our previous findings, this suggests that TRPM4 mediates apoptosis through the mitochondrial pathway under PA treatment.

### 3.5 miR-133a-3p bind to the TRPM4 3′-UTR and inhibit expression

**FIGURE 5:**
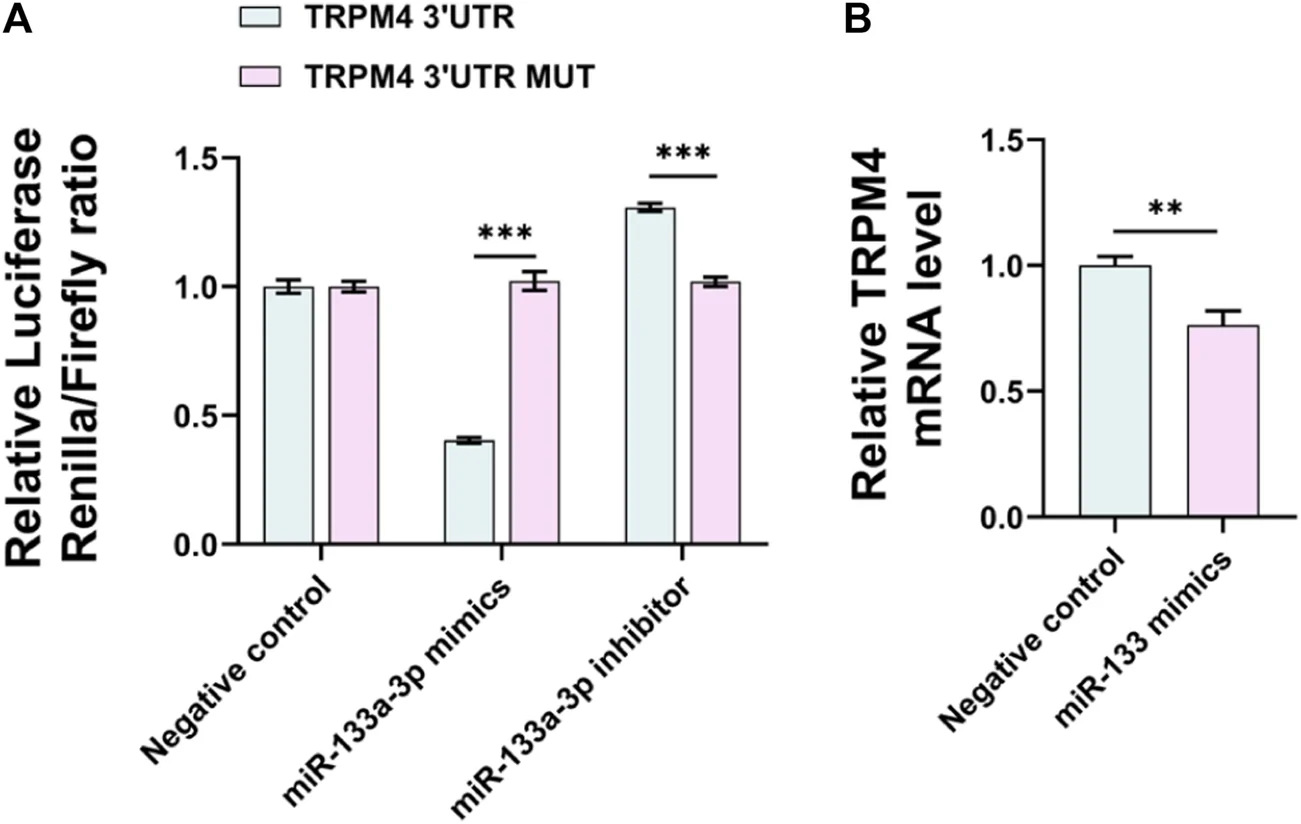
miR-133a-3p can bind to the TRPM4 3′UTR and inhibit TRPM4 expression. **(A)** HEK293T cells were transfected with miR-133a-3p mimics, miR-133a-3p inhibitor, or NC siRNA, and psi-CHECK™-2-target DNA or blank plasmid and firefly/renilla ratio was measured by a dual-luciferase reporter kit. Data are expressed as the mean ± SEM, *n* = 3, ***p < 0.001 vs. TRPM4 3′UTR group. **(B)** qRT-PCR analysis of TRPM4 mRNA expression in HUVECs after transfected with miR-133a-3p mimics. Data are expressed as the mean ± SEM, *n* = 3, **p < 0.01, ***p < 0.001 vs. negative control.

miRNAs adjust the expression of most protein-coding genes, and the same miRNA may inhibit multiple target genes or act on multiple mRNAs simultaneously. Recent researchers have detected that miRNAs play an essential role in lipid metabolism by regulating the metabolism of cholesterol, LDL receptors, fatty acids, etc (Pincu et al., 2023). It has been reported that TRP channels are controlled by some miRNAs, drug blockade of TRPV4 or selective silencing expression in mouse neurons can protect mice from the effects of miR-203b-3p induced scratching behavior. Overexpression of TRPC1 in podocytes can significantly inhibit severe damage to podocytes and disorder of podocyte cytoskeleton caused by miR-135a expression, and astragaloside IV (ASG) inhibited cardiac fibrosis by targeting the miR-135a-TRPM7-TGF-β/Smad pathway (Yang et al., 2017; Wei et al., 2020; De Logu et al., 2023). We utilized the NCBI database to find miRNAs that can regulate TRPM4 and convey vaso-protective effects and predicted that 168 miRNAs might affix to TRPM4 from Target-scan. This consists of miRNAs like miR-1, miR-122, and miR-133, which have been demonstrated to have connections to coronary artery disease (CAD) and could act as a novel biomarker (Šatrauskienė et al., 2021). Interestingly, mimics of miR-133a/b can counteract the cardiac remodeling and atrial arrhythmia signaling induced by ZFHX3-KD (Cheng et al., 2019), in a way similar to the function of TRPM4 in the heart. This implies that there might be a binding potential between TRPM4 and miR-133a. Next, from this prediction, we focus on miR-133a-3p. The miR-133a-3p and TRPM4 interaction was confirmed via a luciferase reporter gene assay. miR-133a-3p overexpression resulted in a marked reduction in luciferase activity, which intensified with the introduction of a miR-133a-3p inhibitor in HEK293T cells (Figure 5A), and the above effects were not found in mutation locations in the TRPM4 3′UTR. In the end, miR-133a-3p reduces TRPM4 mRNA levels by attaching to the 3′UTR region of TRPM4 (Figure 5B). This suggests that miR-133a-3p directly regulates TRPM4 expression by binding to its 3′UTR region.

### 3.6 miR-133a-3p alleviates PA-induced endothelial injury

**FIGURE 6:**
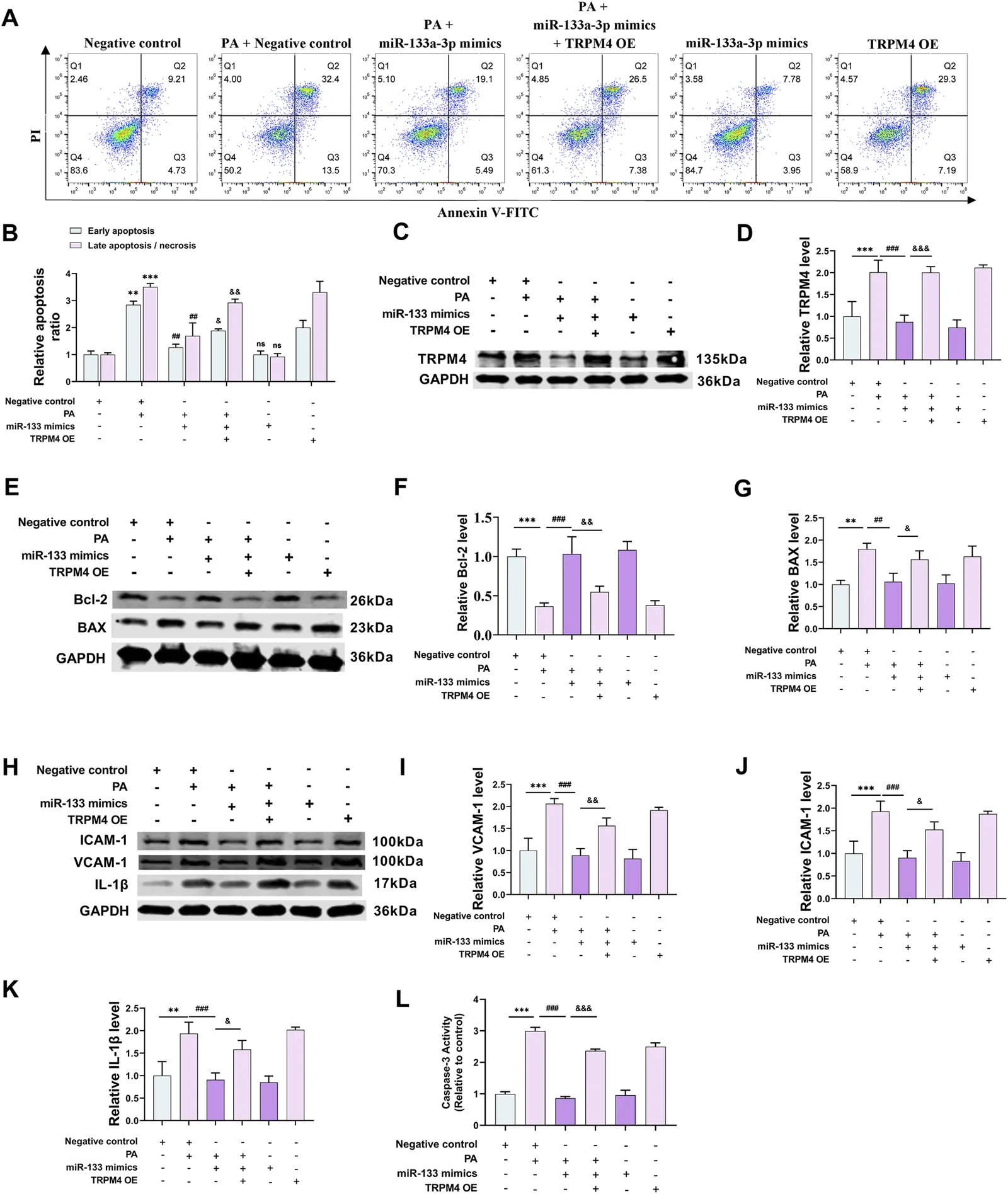
miR-133a-3p alleviates PA-induced endothelial injury. HUVECs were transfected with miR-133a-3p mimics, TRPM4 overexpression plasmid, or negative control and then exposed to 0.2 mM PA or solvent control for 24 h **(A,B)** Annexin V/PI staining assay is used to detect apoptosis (**p < 0.01, ***p < 0.001 vs. NC group; ^##^ *p* < 0.01, vs. NC + PA group; ^&^ *p* < 0.05, ^&&^ *p* < 0.01, vs. miR-133a-3p + PA group). **(C–K)** Western blotting analysis of TRPM4, BAX, Bcl-2, ICAM-1, VCAM-1, and IL-1β protein expression and their quantitative statistics. **(L)** Caspase-3 activity was shown in the different groups. Data are expressed as the mean ± SEM, *n* = 3; **p < 0.01, ***p < 0.001 vs. NC group; ^##^ *p* < 0.01, ^###^ *p* < 0.001vs. NC + PA group; ^&^ *p* < 0.05, ^&&^ *p* < 0.01, ^&&&^ *p* < 0.001vs. miR-133a-3p + PA group.

In order to confirm the protective impact of miR-133a-3p against PA-triggered endothelial damage, we utilized the annexin V/PI apoptosis detection kit in an experimental study. This revealed that the rate of PA-induced cell death was significantly lowered by an increased expression of miR-133a-3p, but this effect was negated by higher levels of TRPM4 (Figures 6A, B). At the same time, the miR-133a-3p reduced the abundance of TRPM4 protein during PA treatment, only to be counteracted by increased expression of TRPM4 (Figures 6C, D). In addition, with PA treatment, cells transfected with miR-133a-3p revealed lower levels of BAX proteins and higher levels of Bcl-2 proteins - an effect that was reversed with the overexpression of TRPM4 (Figures 6E–G). Western blot results verified that the levels of ICAM-1, VCAM-1, and IL-1β proteins were significantly lowered by miR-133a-3p during PA treatment, but these reductions were offset by TRPM4 overexpression (Figures 6H–K). The same results were observed in detecting the activity of caspase-3 (Figure 6L).

Furthermore, TRPM4 overexpression augmented the expression of apoptosis and inflammatory cytokines, yet miR-133a-3p had no significant effects without PA treatment. However, with the addition of PA treatment, miR-133a-3p was able to effectively reduce the expression of these cytokines, indicating its potential as a protective factor against PA-induced endothelial injury. These findings suggest that miR-133a-3p may play a crucial role in mitigating the harmful effects of PA on endothelial cells, and its function is closely linked to the regulation of TRPM4 expression.

### 3.7 miR-133a-3p alleviates PA-induced vascular endothelial injury via the mitochondrial pathway

**FIGURE 7:**
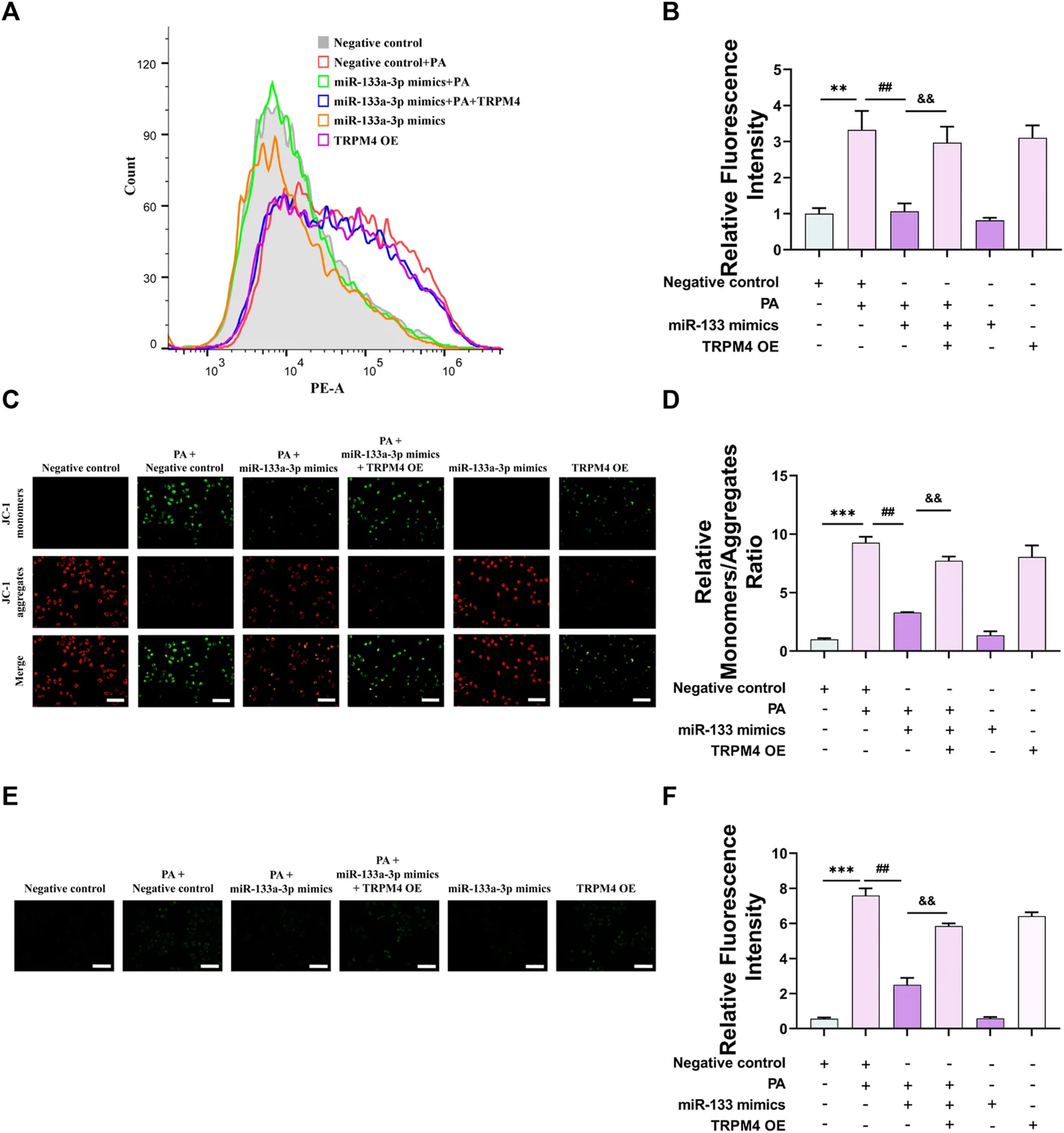
miR-133a-3p alleviates PA-induced vascular endothelial injury via the mitochondrial pathway. HUVECs were transfected with miR-133a-3p mimics, TRPM4 overexpression plasmid, or negative control and then exposed to 0.2 mM PA or solvent control for 24 h **(A, B)** The relative fluorescence intensity of intracellular calcium was displayed by Fluo-4 AM through flow cytometry. **(C)** Fluorescence microscopy analysis of the level of JC-1 monomers (cells with green fluorescence) and J-aggregate forms (cells with red fluorescence). Scale bar, 50 μm. **(D)** The quantified relative monomers/aggregates fluorescence rate. **(E)** Fluorescence microscopy analysis of the level of cellular ROS. **(F)** The quantified relative fluorescence intensity. Scale bar, 50 μm. Data are expressed as the mean ± SEM, *n* = 3, **p < 0.01, ***p < 0.001 vs. NC group; ##*p* < 0.01, vs. NC + PA group; && *p* < 0.01, vs. miR-133a-3p + PA group.

We utilized flow cytometry to probe into the potential role of miR-133a-3p in regulation, by measuring calcium ion fluorescence intensity and quantifying intracellular calcium levels. It was observed that an increase in miR-133a-3p dramatically reduces the fluorescence intensity of calcium ions following PA stimulation, however, this protective effect of miR-133a-3p is weakened by the overexpression of TRPM4 (Figures 7A, B). Next, we investigated mitochondrial membrane potential (∆Ψm) and ROS level in endothelial cells. Following PA stimulation, a transition of the JC-1 probes from red to green fluorescence was detected, leading to a decrease in cell membrane potential levels and onset of early apoptosis. The overexpression of miR-133a-3p mitigates the transition process of JC-1’s red fluorescence and helps in restoring membrane potential levels. However, overexpression of TRPM4 counters the protective effect of miR-133a-3p (Figures 7C, D). Similar outcomes were found when intracellular ROS levels were measured using DCF fluorescent probes (Figures 7E, F).

**FIGURE 8:**
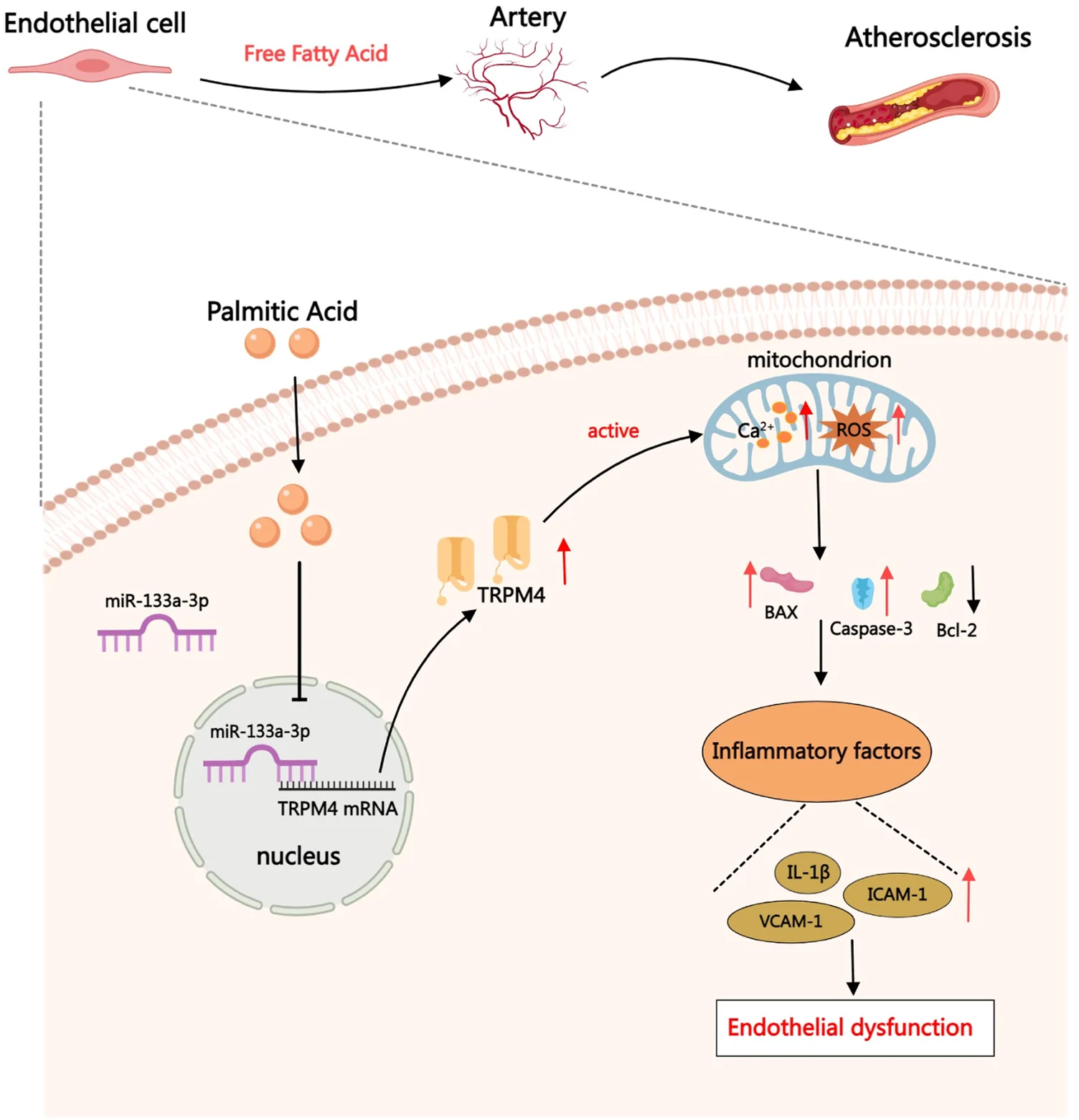
When undergoing therapy with PA, TRPM4’s expression levels elevate, stimulating cell membrane depolarization and leading to an overabundance of calcium. This abundance triggers BAX activation along with other members in the Bcl-2 family, amplifying the generation of PTP. Damage to the mitochondria prompts an accumulation of ROS, which then sparks inflammation and enhances the signaling of inflammatory cytokines. Consequently, monocytes are summoned to join in the inflammatory reaction.

In the chain of events resulting from PA-induced endothelial injury, the expression of miR-133a-3p diminishes. This drop in expression lessens its attachment to TRPM4, causing an increase in TRPM4 expression along with a rise in intracellular calcium ion levels. Furthermore, it also leads to a decrease in mitochondrial membrane potential and ROS levels. By either suppressing the TRPM4 expression level or augmenting miR-133a-3p content, the endothelial damage instigated by PA can be reversed (Figure 8). This has the potential to pave a new path for treating hyperlipidemia patients and could provide a more focused method in handling endothelial dysfunction linked with hyperlipidemia and potentially lessen cardiovascular disease risk in these patients. Moreover, extensive research is needed to uncover the therapeutic possibilities of tweaking miR-133a-3p and TRPM4 in different pathophysiological states involving endothelial dysfunction. This would help in determining the efficacy and safety of such interventions to develop effective treatment strategies for patients.

## 4 Discussion

Atherosclerosis, the formation of fibrofatty lesions in artery walls, is a severe threat to human health. Currently, commonly used therapies for the treatment of AS include lowering plasma cholesterol, anti-platelet aggregation, vasodilation, thrombolytic, etc (Libby et al., 2019; Ruotsalainen et al., 2021). Which are almost all related to the ability of vascular endothelium. Endothelial cells play an irreplaceable role in maintaining tissue blood circulation and material exchange. Endothelial cells monitor molecular transport in plasma through transport and endocytosis, playing a role in adjusting vasodilation, cellular cholesterol efflux, and lipid metabolism. These cells are also pitch in blood clotting, signal transduction, inflammation, and immunity (Mehta and Malik, 2006; Simionescu and Antohe, 2006). Cardiovascular risk factors, such as hyperlipemia and dyslipidemia, cause the molecular machinery of the endothelial cells to respond to shocks by modulating their constitutive activities, which is followed by malfunction and, eventually, injury and apoptosis (Sima et al., 2009). Traditional medications come with several side effects and may not successfully protect the vascular endothelium over the long run (Cook et al., 2007). Therefore, the goal of our investigation is to discover new endothelium protection targets.

Hyperlipidemia has been identified as one of the most significant risk factors for the occurrence of coronary heart disease. Hyperlipidemia-related lipid problems are thought to be one of the etiology of atherosclerotic cardiovascular disease (El-Tantawy and Temraz, 2019). Elevated levels of free fatty acids abnormally can cause an increase in the production of very low-density lipoproteins and speed up the rate of fat deposition within blood vessels. Palmitic acid, which is the most abundant free fatty acid in the body, is highly inflammatory. It is frequently used in the creation of *in vitro* models for lipotoxicity. In the present study, we used PA to cause endothelium injury and carried out further experiments. Consistent with previous studies (Hu et al., 2018; Shi et al., 2019), we described that PA-induced apoptosis promoted ICAM-1, VCAM-1, and IL-1β expression in HUVECs (Figure 1).

TRPM4 is a calcium-activated non-selective cationic channel, and its activation causes the plasma membrane to depolarize, resulting in calcium influx (Guinamard et al., 2010). In patients with various forms of heart disease (e.g., Brugada syndrome, heart block, and congenital long QT syndrome), scientists have identified numerous pathogenic variants in the TRPM4 gene (Amarouch and El Hilaly, 2020). In our earlier research, TRPM4 mediated ATO-induced vascular endothelial injury (Yu et al., 2019). Nevertheless, the specific molecular mechanism by which TRPM4 causes endothelium injury is still unknown. We discovered that TRPM4 total protein was upregulated in response to PA treatment, as was TRPM4 membrane protein level (Figure 2). This indicated that the membrane’s characteristics accomplish the role TRPM4 plays in endothelium injury.

Additionally, using an Annexin V/PI staining kit, we discovered that TRPM4 siRNA could reduce both early apoptosis and late apoptosis/necrosis (Figures 3B, C). Early apoptosis, characterized by DNA fragmentation, is reversible (Geske et al., 2001). Focusing on TRPM4 during the early stage of vascular endothelial injury is advantageous for vascular health because it helps to maintain a healthy condition. Both ICAM-1 and VCAM-1, which are part of the immunoglobulin superfamily, play a crucial role in leukocyte migration into the subendothelial space. Furthermore, they also contribute to the activation of endothelial cells and the development of atherosclerotic plaques, directly promoting inflammatory responses within the vessel wall (Lawson and Wolf, 2009; Troncoso et al., 2021). TRPM4 siRNA exerted anti-inflammatory effects by preventing the expression of ICAM-1 and VCAM-1 (Figures 3I–K). IL-1β, one of the 11 members of the IL-1 family, has become a therapeutic target for a growing range of inflammatory diseases. IL-1β neutralization alleviated the patients’ symptoms quickly and consistently (Dinarello, 2011). TRPM4 knockdown considerably reduced IL-1β levels and may provide therapeutic benefits for a number of cardiovascular illnesses (Figures 3I–L).

We dug deeper into the mechanism by which TRPM4 causes vascular endothelial injury. Numerous TRP family members affect mitochondrial activity and generate excessive ROS, which leads to heart diseases (Hof et al., 2019; Mirbod et al., 2023). The SUR1-TRPM4/NOS2/ROS signaling pathway mediates the inflammatory response in ischemia-reperfusion damage (Maqoud et al., 2022). The mitochondrial apoptotic process depends on the production of PTP and the release of cytochrome c to the cytoplasm. BAX, a crucial component of PTP, was decreased, while Bcl-2, an inhibitor of BAX, was increased (Spitz and Gavathiotis, 2022), suggesting that TRPM4 knockdown prevented apoptosis under PA exposure via the mitochondrial pathway (Figures 3F–H). The recovery of ∆Ψm and the decline in ROS following TRPM4 knockdown in PA-treated conditions served as confirmation of this (Figures 4C–F). Sustained oxidative stress-induced inflammation (Di Lisa et al., 2009; Yuan et al., 2019), accompanied by high levels of ICAM, VCAM, and IL-1β, consistent with our study (Figures 3I–L)). How does TRPM4 modulate the synthesis of mitochondrial PTP? Our research revealed upregulated intracellular Ca^2+^ levels under PA treatment, and TRPM4 knockdown reversed this alteration (Figures 4A, B). Na^+^ was allowed to enter cells upon TRPM4 activation, but Ca^2+^ was excluded entirely. However, Ca^2+^ influx is activated by the depolarization brought on by Na^+^ inflow, following a series of signal transduction within the cell (Guinamard et al., 2010; Ezeani, 2019; Diszházi et al., 2021). Mitochondrial permeability transition can be triggered by Ca^2+^, leading to leakage of mitochondrial contents and loss of the structural and functional integrity of the mitochondria (Fiskum et al., 2003). In summary, mitochondria play a critical role in apoptosis and inflammation progression.

miRNA, a small non-coding RNA, is critical in preserving normal physiological functions within the human body. There are numerous factors such as cell type, environmental conditions, and intracellular target gene expression patterns that shape the function of miRNA (Jusic et al., 2022). These elements can modulate the interaction between miRNA and its specific target genes and also the regulation of subsequent signaling pathways, resulting in a variety of biological outcomes. Moreover, miRNAs are not uniformly expressed across different cell types, and the cell type itself can affect the expression level of miRNAs. Past research indicates that irregular miRNA expression plays a part in steering the progression of a range of cardiovascular diseases. miR-133a-3p affects the progression of atherosclerosis (Libby et al., 2019), cardiac remodeling (Li et al., 2018), myocardial infarction (Paiva and Agbulut, 2017), ischemia-reperfusion injury (Ye et al., 2011). Several studies have demonstrated that miR-133a-3p, which is considered as a candidate marker, is markedly downregulated in obesity and cardiovascular disease (Kim et al., 2020; Abdallah et al., 2022). However, the specific role and mechanism of miR-133a-3p in atherosclerosis remains unclear.

Our research showed that PA caused injury to the endothelial cells, leading to a drop in miR-133a-3p expression. This caused a decrease in the binding to the TRPM4’s 3′UTR region, and increased TRPM4’s expression level, further intensifying the level of vascular endothelial damage. Bcl-2, a well-known anti-apoptotic protein in the Bcl-2 family, prevents apoptosis by constructing heterodimers with BAX. As regards to its expression regulation, there was a reversal in the PA-induced downregulation of Bcl-2 expression with the overexpression of miR-133a-3p. This implies that miR-133a-3p also has a role in enhancing Bcl-2 expression in endothelial cells post hyperlipidemia injury. Similarly, some researchers showed a decrease in miR-133a expression in Parkinson’s cell model. They demonstrated that the overexpression of miR-133a specifically inhibits RAC1, promotes cell growth, and reduces cell apoptosis and autophagy levels (Lu et al., 2020).

miRNA has the ability to bind to the 3′UTR region of target genes, leading to the degradation or inhibition of target gene expression. This ultimately contributes to the regulation of physiological functions in the body. Through bioinformatics prediction analysis, a targeted relationship between miR-133a-3p and TRPM4 was identified. To confirm this relationship, the study conducted *in vitro* experiments and dual fluorescence reporting experiments, which confirmed that miR-133a-3p specifically binds to the predicted target site of TRPM4.

Nevertheless, it is important to note that this study has certain limitations. It fails to explain the sequential relationship between endothelial injury events and the abnormal expression of miR-133a-3p and TRPM4 after the stimulation of endothelial cells by PA. Additionally, the study does not shed light on the influence of miR-133a-3p on the distribution of TRPM4. Further studies are needed to prove this hypothesis in animal models of atherosclerosis.

To sum up, our study demonstrated that interfering TRPM4 expression significantly improved the endothelial function via the mitochondrial pathway under PA treatment. Moreover, miR-133a-3p as a negative regulator of TRPM4 and exerts protective effects on the endothelium. Our study provides a novel potential target for the prevention of endothelial injury and atherosclerosis.

## Data Availability

The original contributions presented in the study are included in the article, further inquiries can be directed to the corresponding authors.
