# Correction: miR-133a-3p/TRPM4 axis improves palmitic acid induced vascular endothelial injury

**DOI:** 10.3389/fphar.2026.1960435

**Published:** 2026-10-15

**Authors:** Yadong Xue, Tingting Tong, Yuyao Zhang, Haijun Huang, Ling Zhao, Hongzhao Lv, Lingzhao Xiong, Kai Zhang, Yuxuan Han, Yuyang Fu, Yongzhen Wang, Rong Huo, Ning Wang, Tao Ban

**Affiliations:** 1 Department of Pharmacology (The Key Laboratory of Cardiovascular Research, Ministry of Education, State Key Laboratory of Frigid Zone Cardiovascular Diseases, Ministry of Science and Technology) at College of Pharmacy, Harbin Medical University, Harbin, China; 2 Department of Anatomy, School of Basic Medical Sciences, Heilongjiang University of Chinese Medicine, Harbin, China; 3 Department of General Surgery, The Fourth Affiliated Hospital of Harbin Medical University, Harbin, China; 4 Heilongjiang Academy of Medical Sciences, Harbin, China; 5 National-Local Joint Engineering Laboratory of Drug Research and Development of Cardio-Cerebrovascular Diseases in Frigid Zone, The National Development and Reform Commission, Harbin, China

**Keywords:** vascular endothelial injury, hyperlipidemia, palmitic acid, TRPM4, MiR-133a-3p

There was a mistake in Figures 3C, 6B as published. The figure legend labeled the Annexin V+/PI+ (Q2) population as “late apoptosis”, which is inaccurate because this population cannot be distinguished from primary necrotic cells. The correct designation should be “late apoptosis/necrosis”. The corrected Figures 3, 6 appear below.

**FIGURE 3:**
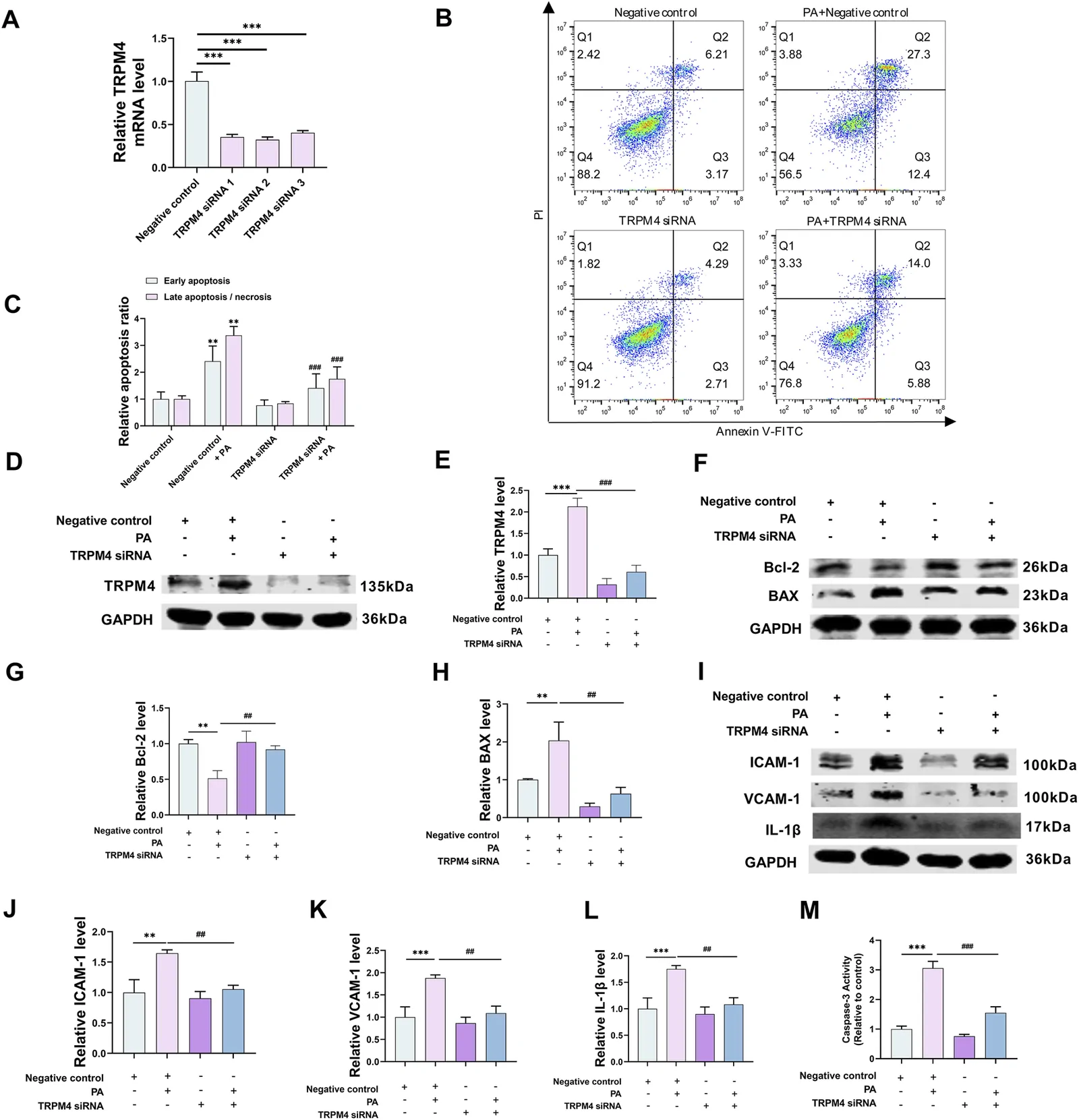
Knockdown of TRPM4 alleviates PA-induced apoptosis and inflammatory cytokines expression. **(A)** HUVECs knockdown TRPM4 efficiency evaluated by qRT-PCR. HUVECs were transfected with TRPM4 siRNA one or its negative control and exposed to PA for 24 h or solvent control for subsequent experiments. **(B,C)** Annexin V/PI staining assay was used to detect apoptosis. **(D–L)** Western blotting analysis of TRPM4, BAX, Bcl-2, ICAM-1, VCAM-1, and IL-1β protein expression and their quantitative statistics. **(M)** Caspase-3 activity was shown in the different groups. Data are expressed as the mean ± SEM, n = 3–4; in each group, **P < 0.01, ***P < 0.001 vs. negative control; ^##^ *P* < 0.01, ^###^ *P* < 0.001 vs. negative control + PA.

**FIGURE 6:**
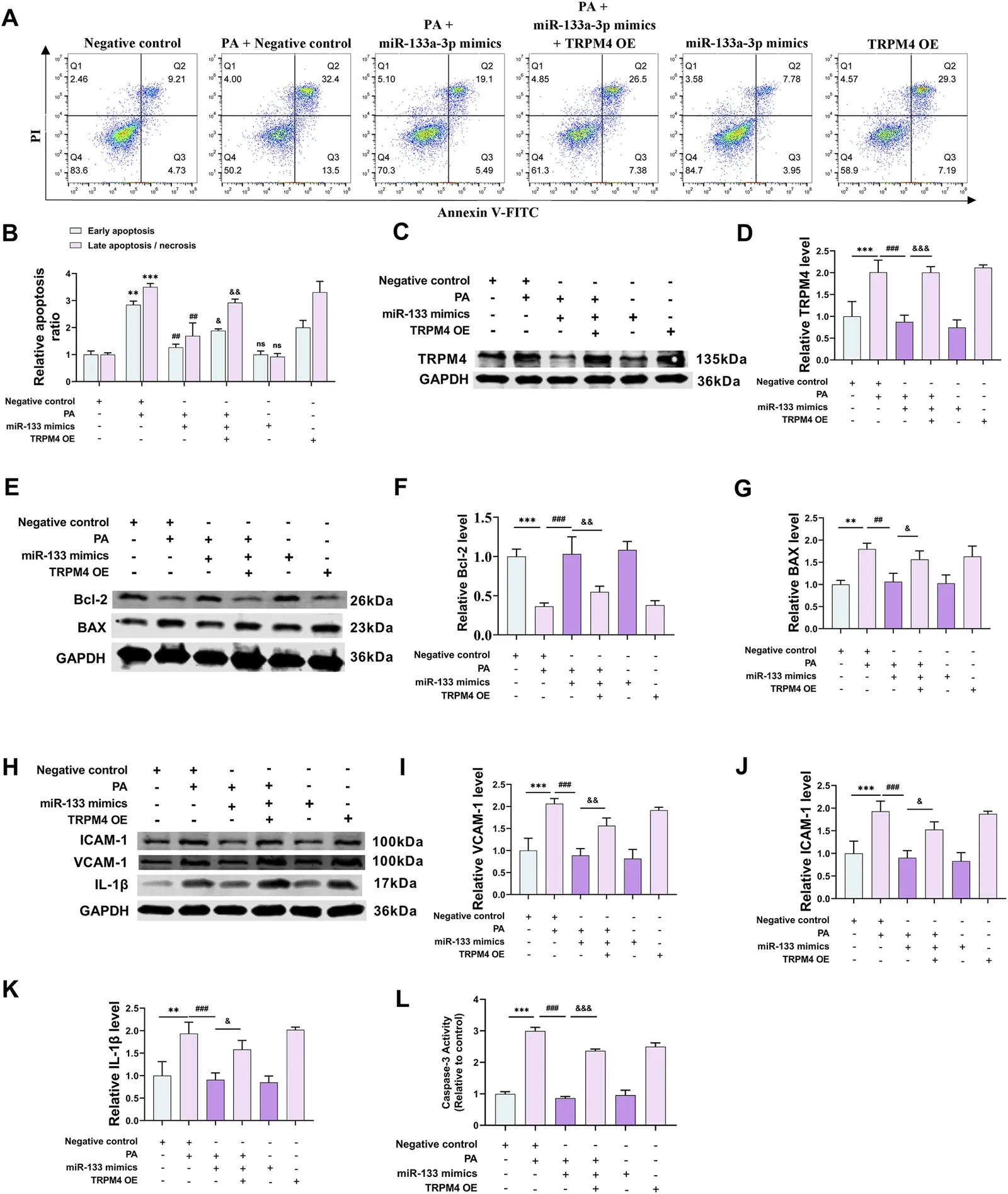
miR-133a-3p alleviates PA-induced endothelial injury. HUVECs were transfected with miR-133a-3p mimics, TRPM4 overexpression plasmid, or negative control and then exposed to 0.2 mM PA or solvent control for 24 h. **(A,B)** Annexin V/PI staining assay is used to detect apoptosis (**P < 0.01, ***P < 0.001 vs. NC group; ^##^ *P* < 0.01, vs. NC + PA group; ^&^ *P* < 0.05, ^&&^ *P* < 0.01, vs. miR-133a-3p + PA group). **(C–K)** Western blotting analysis of TRPM4, BAX, Bcl-2, ICAM-1, VCAM-1, and IL-1β protein expression and their quantitative statistics. **(L)** Caspase-3 activity was shown in the different groups. Data are expressed as the mean ± SEM, n = 3; **P < 0.01, ***P < 0.001 vs. NC group; ^##^ *P* < 0.01, ^###^ *P* < 0.001vs. NC + PA group; ^&^ *P* < 0.05, ^&&^ *P* < 0.01, ^&&&^ *P* < 0.001vs. miR-133a-3p + PA group.

An incorrect Funding statement was provided in the published article. The first sentence incorrectly states that no financial support was received. This has been corrected to: “The author(s) declare that financial support was received for the research and/or publication of this article”.

The term “late apoptosis” was used throughout the text to describe the Annexin V+/PI+ (Q2) population. This is inaccurate because this population cannot be distinguished from primary necrotic cells. The correct designation should be “late apoptosis/necrosis.”

A correction has been made to section **3 Results**, *3.3 Knockdown of TRPM4 alleviates PA-induced apoptosis and inflammatory cytokines expression,* paragraph 1:

“Without PA treatment, TRPM4 knockdown did not affect the apoptosis of HUVECs. Interestingly, PA affected not only early apoptosis but also late apoptosis/necrosis, and TRPM4 knockdown after PA stimulation relieves both early apoptosis and late apoptosis/necrosis (Figures 3B,C)”.

A correction has been made to section **4 Discussion,** paragraph 4:

“Additionally, using an Annexin V/PI staining kit, we discovered that TRPM4 siRNA could reduce both early apoptosis and late apoptosis/necrosis (Figures 3B,C)”.

The original version of this article has been updated.

